# Analysis of Factors Influencing Diagnostic Accuracy of T-SPOT.TB for Active Tuberculosis in Clinical Practice

**DOI:** 10.1038/s41598-017-07785-6

**Published:** 2017-08-10

**Authors:** Lifan Zhang, Xiaochun Shi, Yueqiu Zhang, Yao Zhang, Feifei Huo, Baotong Zhou, Guohua Deng, Xiaoqing Liu

**Affiliations:** 1 Division of Infectious Diseases, Peking Union Medical College Hospital, Chinese Academy of Medical Sciences & Peking Union Medical College, Beijing, China; 2 Clinical Epidemiology Unit, International Epidemiology Network, Chinese Academy of Medical Sciences & Peking Union Medical College, Beijing, China

## Abstract

T-SPOT.TB didn’t perform a perfect diagnosis for active tuberculosis (ATB), and some factors may influence the results. We did this study to evaluate possible factors associated with the sensitivity and specificity of T-SPOT.TB, and the diagnostic parameters under varied conditions. Patients with suspected ATB were enrolled prospectively. Influencing factors of the sensitivity and specificity of T-SPOT.TB were evaluated using logistic regression models. Sensitivity, specificity, predictive values (PV), and likelihood ratios (LR) were calculated with consideration of relevant factors. Of the 865 participants, 205 (23.7%) had ATB, including 58 (28.3%) microbiologically confirmed TB and 147 (71.7%) clinically diagnosed TB. 615 (71.7%) were non-TB. 45 (5.2%) cases were clinically indeterminate and excluded from the final analysis. In multivariate analysis, serous effusion was the only independent risk factor related to lower sensitivity (OR = 0.39, 95% CI: 0.18–0.81) among patients with ATB. Among non-TB patients, age, TB history, immunosuppressive agents/glucocorticoid treatment and lymphocyte count were the independent risk factors related to specificity of T-SPOT.TB. Sensitivity, specificity, PV+, PV−, LR+ and LR− of T-SPOT.TB for diagnosis of ATB were 78.5%, 74.1%, 50.3%, 91.2%, 3.0 and 0.3, respectively. This study suggests that influencing factors of sensitivity and specificity of T-SPOT.TB should be considered for interpretation of T-SPOT.TB results.

## Introduction

WHO reported that 9.6 million people developed TB disease in 2014 worldwide, with a prevalence of 133/100000, and 1.5 million people died of TB^1^.

TB burden is still enormous in China due to the large population. China is one of the 30 countries with high TB burden, and also with high TB/HIV and MDR-TB burden. WHO estimated that China had 0.93 million of new cases of TB in 2014, accounting for 10% of the world. In 2010, the fifth national TB epidemiological survey suggested that the prevalence of active pulmonary TB was 459/100000 in people above 15 years old^2^.

Diagnosis of TB remains a challenge in developing countries. For commonly used diagnostic tools, the sensitivity of acid-fast microscopy is low, the turnaround time of culture is long, and histopathology examinations are expensive andusually invasive. All these methods have limitations to diagnose extrapulmonary TB. Over the last century, tuberculin skin test (TST) was widely used. Despite its simple procedure and low-cost, the specificity of TST is compromised by cross-reactivity with BCG vaccine and non-tuberculosis mycobacterium, and its sensitivity is not stable, for instance, in immunocompromised populations^3, 4^.

As a new immunodiagnostic method of tuberculosis infection, T-cell–based interferon-gamma release assays (IGRA) is now widely recognized and has been developed as a sensitive, specific and rapid immunodiagnostic test for TB infection. It detects interferon-γ secreted by T cells in response to specific antigens from Mycobacterium tuberculosis (MTB), commonly 6kD early secretory antigenic target-6 (ESAT-6) and 10kD culture filtrate protein-10 (CFP-10). Studies confirmed that IGRA correlated better with time and intensity of exposure to active MTB patients compared to TST, independent of BCG-vaccination status^5, 6^.

Many studies confirmed that IGRA was sensitive and specific for diagnosing ATB in low and intermediate epidemic countries, and was unaffected by prior BCG-vaccination^7^. Some studies were done for evaluating the diagnostic value of IGRA^8–10^. However, the data from the low- and middle-income countries was varied and limited, especially in China where the neonatal BCG vaccination was included in the national immunization system^11^. In addition, the data from large-sample cohort studies to evaluate factors influencing the accuracy of IGRA for diagnosing ATB was limited.

In this prospective cohort study, we sought to evaluate the performance of T-SPOT.TB in the diagnosis of ATB in HIV-negative hospitalized Chinese adult patients and to analyze the factors related to its sensitivity and specificity.

## Methods

### Participants

All patients with suspected ATB who were admitted into a tertiary hospital, Peking Union Medical College Hospital (PUMCH), for further evaluation between May 2005 and April 2013 were considered. ATB was suspected if (1) a patient presented with clinical manifestations consistent with pulmonary or extrapulmonary TB, and (2) routine laboratory and radiological examination suggested diagnosis of TB or could not exclude TB. The inclusion criteria also included age ≥16 years, HIV negative, and receiving tuberculosis treatment within 4 weeks. The exclusion criteria included invalid T-SPOT.TB test and indeterminate T-SPOT.TB results. This study was approved by the Ethics Committee of PUMCH and all subjects gave written informed consent. All methods were performed in accordance with the relevant guidelines and regulations.

### T-SPOT.TB assays

Four milliliters of peripheral blood was collected from each patient. Specific T cell responses to RD1 encoded antigens were detected by T-SPOT.TB (Oxford Immunotec, Abingdon, UK) that was performed within 6 hours from sample collection by laboratory personnel blinded to patients’ clinical data. T-SPOT.TB utilized AIM-V (GIBCO™ AIM V Medium liquid, Invitrogen, US.) as negative control, PHA as positive control, and ESAT-6 and CFP-10 as specific antigens, respectively. PBMC obtained from each subject were plated (2.5 × 10^5^ per well) on a plate precoated with the antibody against interferon γ. Plates were incubated 16–18 h at 37 °C in 5% carbon dioxide. After incubation, wells were developed with a conjugate against the antibody used and an enzyme substrate. Spot-forming cells (SFCs) were counted with an automated ELISpot reader (AID-ispot, Strassberg, Germany), each SFC represented an antigen-specific T cell secreting interferon γ. The response was considered positive when the antigen well contained 6 or more spots and had twice the number of spots than the negative control well. The background number of spots in negative control well for PBMC should be less than 10 spots.

### Diagnosis

Attending physicians performed diagnostic work-up as part of routine clinical practice. Information on socio-demographic, clinical, radiologic and microbiological data was extracted from patients’ medical records by researchers blinded to the T-SPOT.TB results. Each case was classified into one of the predefined categories (Table 1, Supplementary Table), based on clinical, radiological, microbiological information and response to anti-TB therapy. For a patient didn’t have a confirmed diagnosis at discharge, the research group conducted an active follow-up at 4 weeks, 8 weeks, 12 weeks, and 24 weeks respectively after discharge by calling the patient, to obtain the final diagnosis. Patients who still had an unclear diagnosis at 24 week follow-up were classified as clinically undeterminate. The classification was adjudicated by two independent physicians blinded to the results of T-SPOT.TB. If there was disagreement on the final diagnosis, a chief physician was referred.

**Table 1:** Categorization of the Study Population.

Diagnostic Category	Criteria
Bacteriologically/histologically-confirmed TB	Suggestive clinical manifestations such as fever, cough and pectoralgia and radiologic findings, and smear acid-fast stain or culture positive for MTB, typical histologic changes (caseous necrosis, epithelioid granulomatous, etc.)
Clinically diagnosed TB	Clinical manifestations (fever, cough, chest pain, night sweats, weight loss, etc.), laboratory results and radiologic features highly suggestive of tuberculosis, and appropriate response to anti-TB therapy
Clinically indeterminate	A final diagnosis of tuberculosis was neither confirmed nor reliably excluded
Active TB excluded	All microbiological samples of smear acid-fast stain and culture negative, a definite alternative diagnosis identified, and effective treatment of the primary disease

### Statistical analysis

Continuous data of normal distribution were expressed as means ± standard deviation (SD), while data without a normal distribution were described as median and IQR. Categorical data were expressed in percentages. Kolmogorov-Smirnov test was used to examine normal distribution. Independent Samples t-test was used for the continuous variables normally distributed, and Mann-Whitney U test was used for the variables without normal distribution. Categorical variables were compared with chi-squaretest. Factors associated with sensitivity and specificity including previous history of TB, use of glucocorticoids/immunosuppressants, serous effusion, and age were evaluated with stepwise multivariable logistic regression models (inclusion threshold P < 0.05, exclusion threshold P > 0.1).

Diagnostic performance of T-SPOT.TB was evaluated with calculated sensitivity, specificity, positive predictive value (PPV), negative predictive value (NPV), positive likelihood ratio (PLR) and negative likelihood ratio (NLR) from 2 × 2 table. Statistical analyses were performed with SPSS 16.0 (SPSS Inc, USA).

## Results

**Figure 1:**
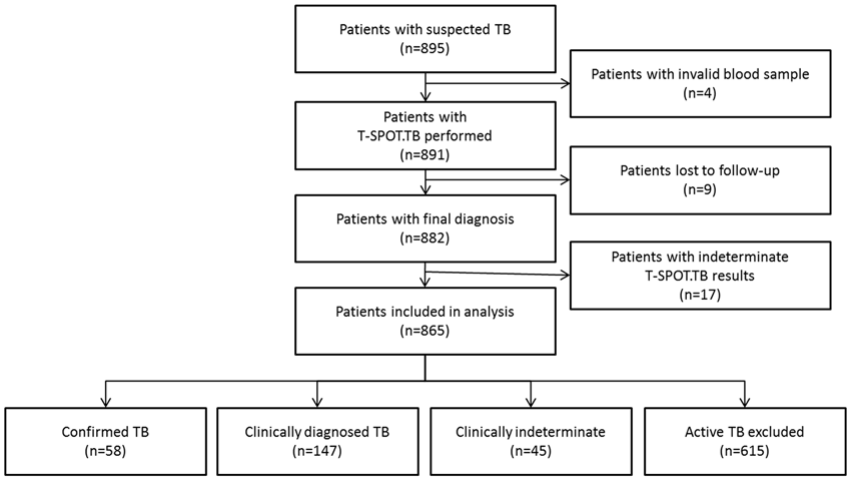
Flowchart of enrollment of patients with suspected tuberculosis.

Among 895 HIV-negative hospitalized patients with suspected TB, 13 patients were excluded from analysis due to invalid blood samples for T-SPOT.TB testing (n = 4) or lost to follow-up before week 24 after enrollment (n = 9). 17 out of 882 patients (1.9%) got indeterminate T-SPOT.TB results, and 865 patients were included into analysis (Fig. 1).

### Demographic and clinical characteristics of study participants

Among the included 865 patients, the median age was 47 years (range 16–91) and 423 (48.9%) were female (Table 2). 205 (23.7%) patients had ATB, including 58 (28.3%) with bacteriologically or histologically confirmed TB and 147 (71.7%) with clinically diagnosed TB. Eighty (39.0%) were pulmonary TB, and 125 (61.0%) were extrapulmonary TB. The most common sites of TB were lungs (31.6%), followed by pleura (11.1%), peritoneum (9.1%) and lymph node(7.1%). 194 (22.4%) had serous effusion (including pleural, peritoneal, pericardial effusion). At the end of study follow-up, ATB was excluded in 615 patients (71.7%), who had other infections (26.2%), autoimmune diseases (28.8%), malignancy (17.6%), and other diseases (27.5%). Forty-five (5.2%) patients were clinically indeterminate.

**Table 2:** Demographic, clinicalcharacteristics, and laboratory examinations of patients included.

	Bacteriologically/histologically-confirmed TB	Clinically diagnosed TB	Clinically indeterminate	Active TB excluded	Total
Total, n (%)	58(6.7)	147(17.0)	45(5.2)	615(71.1)	865
Median age [IQR], y	39[26–65]	48[33–60]	56[34–65]	47[30–61]	47[30–61]
Men, n (%)	27(46.6)	76(51.7)	23(51.v1)	297(48.3)	423(48.9)
Serositis effusion, n (%)	14(24.1)	55(37.4)	12(26.7)	113(18.4)	194(22.4)
Pleural	4	20	4	47	75
Peritoneal	3	9	1	11	22
Pericardial	1	8	2	16	29
Polyserous	6	18	5	39	68
Use of corticosteroids or immunosuppressive agents, n (%)	14(24.1)	14(9.5)	7(15.6)	97(15.8)	132(15.3)
History of TB or radiological features suggestive of previous TB, n (%)	9(15.5)	35(22.4)	4(8.9)	81(13.2)	127(14.7)
Median Lymphocyte count [IQR], cells/mm^3^	1280[610–1780]	1320[920–1810]	1140[810–1770]	1400[890–1930]	1360[880–1900]

### Impact factors of the diagnostic accuracy of T-SPOT.TB test

We analyzed the possible factors influencing the diagnostic accuracy of T-SPOT.TB, including sex, age, serositis, a history of TB or radiological features suggestive of previous TB, application of immunosuppressive agents or glucocorticoid and lymphocyte count.

**Figure 2:**
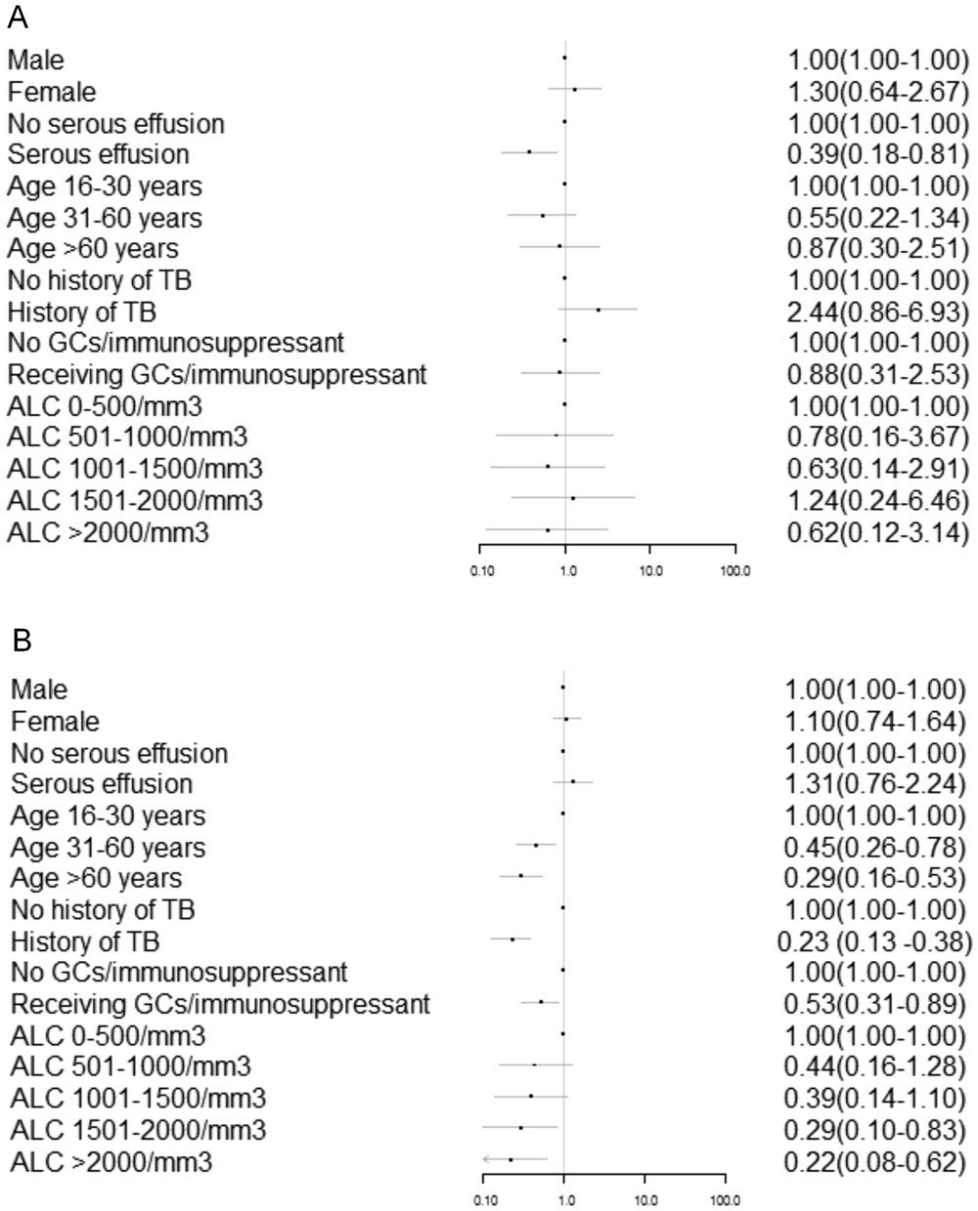
The factors which influence the sensitivity (**A**) and specificity (**B**) of T-SPOT.TB. GCs: glucocorticoids; ALC: absolute lymphocyte count.

Multivariate logistic regression analysis showed that serous effusion was an independent risk factor related to the sensitivity of T-SPOT.TB (OR = 0.39, 95% CI: 0.18–0.81). Age (OR = 0.45, 95% CI: 0.26–0.78, for 31–60 age group; OR = 0.29, 95% CI: 0.16–0.53, for > 60 age group), history of TB or radiological features suggestive of previous TB (OR = 0.23, 95% CI: 0.13–0.38), application of immunosuppressive agents or glucocorticoid (OR = 0.53, 95% CI: 0.31–0.89) and lymphocyte count (OR = 0.29, 95% CI: 0.10–0.83, for 1501–2000/mm^3^; OR = 0.22, 95% CI: 0.08–0.62 for >2000 mm^3^) were the independent risk factors related to the specificity of T-SPOT.TB test (Fig. 2).

### Diagnostic value of T-SPOT.*TB* test on PBMC for active tuberculosis

Among fifty-eight patients with bacteriologically or histologically confirmed tuberculosis, 55 had positive T-SPOT.TB with the sensitivity of 94.8% (95% CI: 86.5–98.9). 106 out of 147 patients with clinically diagnosed tuberculosis had positive T-SPOT.TB with the sensitivity of 72.1% (95% CI: 64.1–79.2), which was significantly lower than that in cases with confirmed tuberculosis (P < 0.001). With the two groups combined, the overall sensitivity of T-SPOT.TB in patients with ATB was 78.5% (161/205, 95% CI: 72.3–84.0). The sensitivity of T-SPOT.TB assay in patients with pulmonary TB appeared higher than that in patients of extrapulmonary TB (85.0% vs. 74.4%, P = 0.071).

Among 615 patients excluded ATB, 456 had negative T-SPOT.TB results, and the overall specificity was 74.1% (95% CI: 70.5–77.6).

Predictive value (PV) and likelihood ratio (LR) for T-SPOT.TB assay was calculated in 820 patients with the definitive diagnosis. The overall PPV, NPV, PLR and NLR of T-SPOT.TB for diagnosis of ATB were 50.3% (95% CI: 44.7–55.9), 91.2% (95% CI: 88.4–93.5), 3.04 (95% CI: 2.61–3.54) and 0.29 (95% CI: 0.22–0.38), respectively. Diagnostic parameters in subgroups are shown in Table 3.

**Table 3:** Sensitivity, specificity, PPV, NPV of ELISPOT (T-SPOT.TB) for diagnosis of active tuberculosis.

	N	Sensitivity % [95% CI]	Specificity % [95% CI]	PPV % [95% CI]	NPV % [95% CI]	PLR [95% CI]	NLR [95% CI]
Diagnosis of active TB	205	78.5[72.3–84.0]	74.1[70.5–77.6]	50.3[44.7–55.9]	91.2[88.4–93.5]	3.04[2.61–3.54]	0.29[0.22–0.38]
Bacteriologically/histologically -confirmed TB	58	94.8[85.6–98.9]	74.1[70.5–77.6]	25.7[20.0–32.1]	99.4[98.1–99.9]	3.67[3.17–4.25]	0.07[0.02–0.21]
Highly probable TB	147	72.1[64.1–79.2]	74.1[70.5–77.6]	40.0[34.1–46.2]	91.8[89.0–94.0]	2.79[2.36–3.30]	0.38[0.29–0.49]
Pulmonary TB	80	88.0[70.0–93.4]	74.1[70.5–77.6]	35.6[29.7–42.0]	97.4[95.6–98.7]	3.40[2.92–3.96]	0.16[0.09–0.28]
Extrapulmonary TB	125	74.4[65.8–81.8]	74.1[70.5–77.6]	36.9[30.9–43.2]	93.4[90.9–95.5]	2.88[2.43–3.41]	0.35[0.26–0.47]
Serous effusion							
No	638	83.8[76.5–89.6]	73.1[69.0–76.9]	45.9[39.5–52.2]	94.3[91.6–96.4]	3.12[2.65–3.67]	0.22[0.15–0.33]
Yes	182	68.1[55.8–78.8]	78.8[70.1–85.9]	66.2[54.0–77.0]	80.2[71.5–87.1]	3.21[2.17–4.74]	0.40[0.28–0.58]
Age 16–30	216	83.3[70.7–92.1]	87.0[80.9–91.8]	68.2[55.6–79.1]	94.0[88.9–97.2]	6.43[4.24–9.75]	0.19[0.11–0.31]
31–60	390	74.5[64.7–82.8]	74.3[68.9–79.2]	49.3[41.0–57.9]	89.7[85.1–93.2]	2.90[2.31–3.64]	0.34[0.24–0.48]
≥61	214	81.1[68.0–90.6]	60.9[52.9–68.5]	40.6[31.1–50.5]	90.7[83.6–95.5]	2.07[1.64–2.62]	0.31[0.17–0.55]
History of TB/radiological features suggestive of previous TB							
No	697	76.1[68.8–82.4]	79.4[75.7–82.8]	53.0[46.4–59.5]	91.6[88.7–93.9]	3.69[3.06–4.45]	0.30[0.23–0.40]
Yes	123	88.1[74.4–96.0]	39.5[28.8–51.0]	43.0[32.4–54.2]	86.5[71.2–95.5]	1.46[1.18–1.79]	0.30[0.13–0.72]
Application of immunosuppressive agents or glucocorticoid							
No	695	78.5[71.7–84.3]	75.3[71.3–79.0]	52.1[45.9–58.2]	91.1[88.0–93.6]	3.18[2.68–3.76]	0.29[0.21–0.38]
Yes	125	78.6[59.1–91.7]	68.0[57.8–77.2]	41.5[28.1–55.9]	91.7[82.7–96.9]	2.46[1.73–3.49]	0.31[0.15–0.65]
Lymphocyte counts 0–1500	459	77.4[69.0–84.4]	78.8[74.0–83.1]	57.5[49.6–65.1]	90.4[86.4–93.5]	3.65[2.91–4.59]	0.29[0.21–0.40]
1501–2000	177	84.2[68.8–94.0]	69.8[61.4–77.3]	43.2[31.8–55.3]	94.2[87.8–97.8]	2.79[2.09–3.72]	0.23[0.11–0.48]
>2001	175	75.0[57.8–87.9]	66.9[58.4–74.7]	37.0[26.0–49.1]	91.2[83.9–95.9]	2.27[1.67–3.07]	0.37[0.21–0.67]

PPV: Positive predictive value; NPV: Negative predictive value; PLR:Positive likelihood ratio; NLR: Negative likelihood ratio.

**Figure 3:**
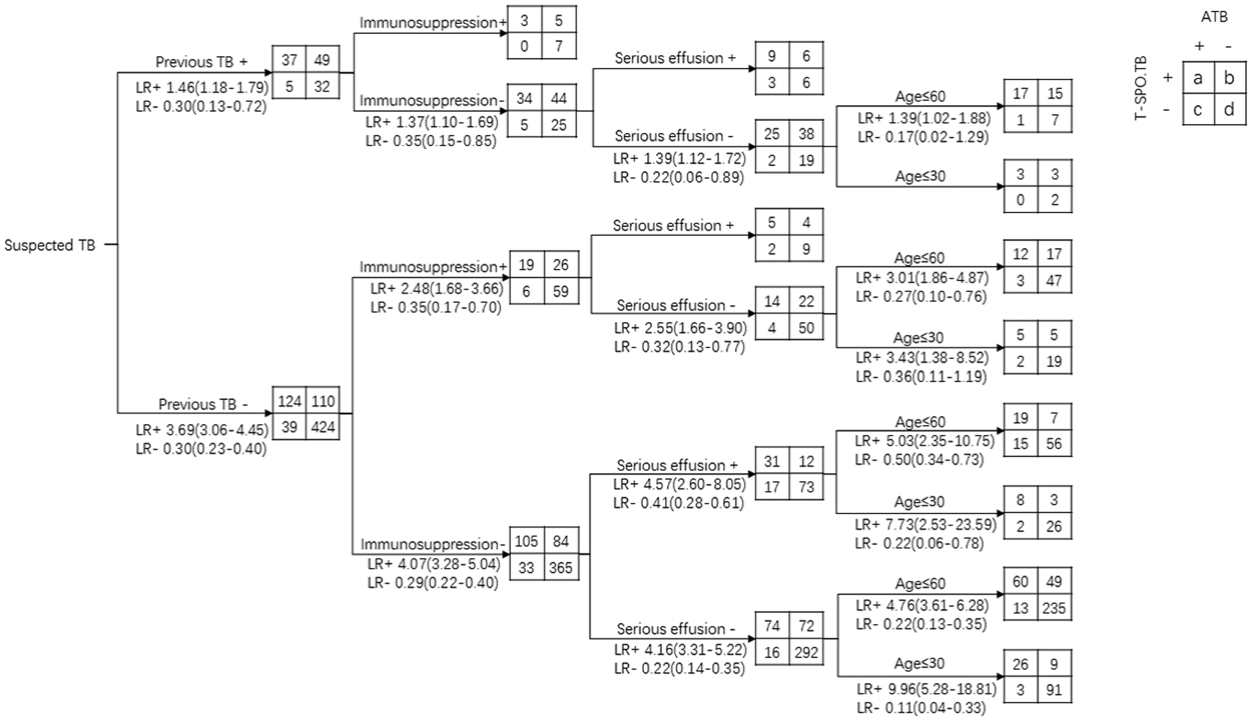
Diagnostic value of T-SPOT.TB of different influencing factors.

We stratified the 820 subjects according to influencing factors including pevious history of TB, immunocompromised condition, serous effusion and age, and drew the diagnostic tree (Fig. 3). For subgroups with more than 30 subjects, PLR and NLR were calculated. Diagnostic value of T-SPOT.TB was high in patients without previous history of TB, no use of immunosuppressant/glucocorticoid, without serous effusion, and under 60 years old, with PLR of 4.76 (95% CI: 3.61–6.28), NLR of 0.22 (95% CI: 0.13–0.35), and especially in patients under 30 years old, with PLR of 9.96 (95% CI: 5.28–18.81), and NLR of 0.11 (95% CI: 0.04–0.33).

### Frequencies of MTB antigen-specific IFN-γ secreting T cells in patients with suspected TB

For 205 patients with ATB, the median count of antigen-specific IFN-γ secreting T cells of T-SPOT.TB was 412 SFCs/10^6^ PBMC (IQR: 116–964). The frequencies of T cells in patients with confirmed tuberculosis was significantly higher than those of clinically diagnosed cases (600 SFCs/10^6^ PBMC, IQR: 276–1060 vs. 280 SFCs/10^6^ PBMC, IQR: 87–942, P = 0.021).

159 out of 615 patients excluded ATB had positive T-SPOT.TB, the median frequencies of T cells were 108 SFCs/106 PBMC (IQR: 48–304), which was significantly lower than those with ATB (P < 0.001). There was no significant difference between the frequencies of T cells specific for ESAT-6 and CFP-10 in all groups.

## Discussion

This large-scale prospective cohort study evaluated the utility of T-SPOT.TB for supporting diagnosis of ATB in HIV-negative hospitalized adult population in a national tertiary medical center in China. Particularly, we analyzed the risk factors related to the diagnostic accuracy of T-SPOT.TB. PUMCH is one of the national referral centers for complex and rare disorders. Most patients had gone to many hospitals for diagnosis and treatment before being admitted to our department. In PUMCH, nearly one-fourth of patients with fever of unknown origin (FUO) were finally diagnosed with ATB^12, 13^. Only one-third of patients with ATB presenting as FUO were microbiologically diagnosed^14^. In this situation, validating simple and rapid tests such as T-SPOT.TB is valuable.

In this study, serous effusion was identified as an independent risk factor related to the sensitivity of T-SPOT.TB. Age, history of TB or radiological features suggestive of previous TB, application of immunosuppressive agents or glucocorticoid and lymphocyte count were the independent risk factors related to the specificity of T-SPOT.TB test. Studies on factors influencing the sensitivity of IGRAs are limited. A study in Vietnam analyzed the false-negative results by QFT-GIT in 19 smear-positive/culture-confirmed TB patients^15^, and demonstrated that a majority of ATB patients showing negative IGRA results did not regain sufficient levels of immune responsiveness despite successful treatment. The authors concluded that insufficient levels of immune responsiveness might impact the sensitivity of IGRAs. Another study of 172 confirmed TB patients in Tanzania suggested that cigarette smoking was associated with false negative QFT-GIT results by logistic regression analysis adjusted for sex, age, HIV and alcohol consumption (OR 17.1, 95% CI: 3.0–99.1, p < 0.01)^16^. Furthermore, it was reported that low peripheral lymphocyte counts and CD4 count in combination with high/normal CD8 count were associated with false-negative results of IGRAs in patients with microbiologically confirmed ATB^17, 18^. It was also reported that aging, emaciation, HIV co-infection and HLA genotype showed significant associations with IGRA negativity in patients with culture-confirmed pulmonary TB^19^.

Our previous study^20^, which detected T-SPOT.TB in patients with tuberculous serositis showed that the sensitivity of T-SPOT.TB on serous fluid and PBMC were 91.9% and 73.0%, respectively. The INF-γ-secreting T-cells were concentrated five times in serous fluid than in blood. Studies in Taiwan^21^ and Europe^22^ showed consistent results. A study with application of QFT-G in diagnosis of tuberculous serositis also reported similar results^23^. A possible reason should be migration of MTB antigen-specific T cells from peripheral blood into serous cavity with the expression of intercellular adhesion molecule, resulting in the lower sensitivity of T-SPOT.TB on PBMC^24^.

IGRA is based on the cellular immune response induced by MTB-specific antigens. Theoretically, it is possible to get positive results of IGRAs when infected with the MTB, including ATB, LTBI and TB history. When diagnosing ATB, IGRAs may give “false-positive” results in patients with LTBI and TB history. A study in China showed that clinical factors, including age (≥46 years) and TB history, were independent risk factors for false-positive outcomes of T-SPOT.TB^25^, which was similar to our findings. In our study, the history of TB/radiological features suggestive of previous TB were independent risk factor of specificity, indicating that specific T-cell reaction may still exist after the recovery of ATB. Furthermore, age and application of immunosuppressive agents/glucocorticoid also significantly influenced the specificity of T-SPOT.TB. The higher positive results of T-SPOT.TB likely reflected the higher proportion of LTBI in patients with these two “proxy” factors^26^. Interestingly, we found that the lymphocyte count might also affect the specificity of T-SPOT.TB in diagnosing ATB, and the results were more likely to be positive when the lymphocyte counts were more than 1500 cells/mm3. A study in Japan demonstrated that the sensitivity decreased significantly with decreasing peripheral lymphocyte count for T-SPOT.TB in patients with microbiologically confirmed active pulmonary TB, and the patients were more likely to have negative results of T-SPOT.TB when lymphocyte counts were less than 500 cells/mm^3^ ^17^.

While the low specificity and positive PV would decrease the utility of T-SPOT.TB in the diagnosis of ATB in high epidemic regions, this study showed that the NPV was high (91.2%), which indicated that the negative result might be valuable to exclude ATB. In high epidemic region, TB is a common cause of FUO and often need to be included in the differential diagnosis. Studies have shown that 21.8%-24.8% of the patients who are admitted to tertiary general hospitals because of FUO are finally diagnosed with ATB per year with the average diagnosing time of 19 weeks^12, 13^. Based on this study, a negative T-SPOT.TB provided an efficient way for excluding ATB in some patients, preventing the unnecessary application of anti-TB drugs and adverse effects of them^27^.

In this study, the sensitivity of T-SPOT.TB for diagnosing ATB was 78.5%, while the sensitivity of T-SPOT.TB for diagnosing confirmed TB was 94.8% and the sensitivity of T-SPOT.TB for diagnosing clinically diagnosed TB was 72.1%. This finding was similar to the result (78.2%) of another study by Qiu, Y *et al*. performed in three TB specialized hospitals in China^28^. Our result was a little lower than the result (85%) of Wang, L *et al*. conducted in a general hospital in western China^29^. In our study, the specificity of T-SPOT.TB for diagnosing TB was 74.1%, which was lower than the results of Qiu, Y (91.1%) and Wang, L (85.1%). A multicentre epidemiological investigation about latent TB infection (LTBI) suggested that the positivity rate of IGRA (QFT-GIT) was 18.9% in rural China^30^, which meant that nearly 20% of the general population in China had LTBI. T-SPOT.TB was positive in 25.9% patients with non-TB in our study, which was higher than the national epidemiological data. We speculate that it was because the study subjects were all inpatients who perhaps were with higher risk of LTBI, and the non-TB group included infections due to other pathogens, rheumatic and immune diseases, and malignant tumor and so on, and 15.8% of these patients had taken glucocorticoid/immunosuppressant, 13.2% of these patients had previous TB/radiological findings of old TB. All these would lead to a positive result of T-SPOT.TB.

When patients were stratified by factors that influence diagnostic performance of T-SPTO.TB, T-SPOT.TB showed the highest diagnostic value in patients without previous history of TB, no use of immunosuppressant/glucocorticoid, without serous effusion, and under 30 years old, with PLR of 9.96 and NLR of 0.11. Therefore, for patients with the above condition, T-SPOT.TB might be more valuable for assisting diagnosis of ATB and excluding ATB in clinical pactice.

As subjects of this study were all HIV-negative patients, results of this study should not apply to HIV/AIDS patients.

One limitation of this study was that many patients with ATB were clinically diagnosed without culture confirmation, which reflects real clinical situation. It would likely underestimate or overestimate the accuracy of T-SPOT.TB for diagnosing ATB. However, all efforts were made to follow up patients who do not have pathogen confirmed TB to make the diagnosis as accurate as possible. Secondly, as the study site is a national center for diagnosis and treatment of complex diseases, the spectrum of disease is expected to be different from TB specific hospitals, and peripheral health care centers. Selection bias may exist and suggest caustions when generalizing the results.

In conclusion, this study showed a moderate sensitivity and specificity of T-SPOT.TB for diagnosis of ATB in a high-burden country. Serous effusion was the independent risk factor to a lower sensitivity, while age (>30 yr), history of tuberculosis/radiological features suggestive of previous tuberculosis, application of immunosuppressive agents or glucocorticoid and lymphocyte counts (>1500 cells/mm^3^) were the independent risk factors to a lower specificity of the T-SPOT.TB test.

## Electronic supplementary material

Supplementary table
