# Supplementary material for: Analysis of Factors Influencing Diagnostic Accuracy of T-SPOT.TB for Active Tuberculosis in Clinical Practice
Source: Sci Rep. 2017 Aug 10;7:7764. doi: 10.1038/s41598-017-07785-6 (PMC5552688; doi:10.1038/s41598-017-07785-6)
Supplement: Supplementary file 1 — Supplementary table [file 41598_2017_7785_MOESM1_ESM.pdf]

Analysis of Factors Influencing Diagnostic Accuracy of T-SPOT.TB for Active Tuberculosis in Clinical Practice

Lifan Zhang[1,2#], Xiaochun Shi[1#], Yueqiu Zhang[1], Yao Zhang[2], Feifei Huo[1], Baotong Zhou[1], Guohua Deng[1], Xiaoqing Liu[1,2]*

[1]Division of Infectious Diseases, Peking Union Medical College Hospital, Chinese Academy of Medical Sciences& Peking Union Medical College

[2]Clinical Epidemiology Unit, International Epidemiology Network, Chinese Academy of Medical Sciences&Peking Union Medical College

*Corresponding Author: Xiaoqing Liu. E-mail: liuxq@pumch.cn.

# Theses authors contributed equally to this manuscript.

Supplementary table. Diagnostic Criteria of extra-pulmonary TB including pleural TB, peritoneal TB, and TB meningitis.

Category	Criteria
Tuberculous pleuritis	
Culture-confirmed	Acid-fast stain or culture positive for MTB of the pleural effusion, OR typical histological changes of the pleura (caseousnecrosis, epithelioid granuloma, etc.) AND suggestive clinical and radiologic findings of tuberculous pleuritis.
Highly probable	Clinical manifestations, laboratory results and radiologic features highly suggestive of tuberculous pleuritis AND appropriate response to anti-TB therapy.
Clinically indeterminate	A final diagnosis was none of the above but tuberculous pleuritis couldn’t be excluded.
Tuberculous peritonitis	
Culture-confirmed	Acid-fast stain or culture positive for MTB of the ascites, OR typical histological changes of the peritoneal (caseousnecrosis, epithelioid granuloma, etc.) AND suggestive clinical and radiologic findings of tuberculous peritonitis.

Highly probable	Clinical manifestations, laboratory results and radiologic features highly suggestive of tuberculous peritonitis AND appropriate response to anti-TB therapy.
Clinically indeterminate	A final diagnosis was none of the above but tuberculous peritonitis couldn’t be excluded.
Tuberculous meningitis	
Culture-confirmed	Acid-fast stain or culture positive for MTB of the cerebrospinal fluid OR typical histological changes of the meninges (caseousnecrosis, epithelioid granuloma, etc.) AND suggestive clinical and radiologic findings of tuberculous meningitis.
Highly probable	Clinical manifestations, laboratory results and radiologic features highly suggestive of tuberculous meningitis AND appropriate response to anti-TB therapy.
Clinically indeterminate	A final diagnosis was none of the above but tuberculous meningitis couldn’t be excluded.
